# Recovery and characterization of cellulose microfibers from fallen leaves and evaluation of their potential as reinforcement agents for production of new biodegradable packaging materials

**DOI:** 10.1002/fsn3.4439

**Published:** 2024-09-02

**Authors:** Sudenur Celik, Gozde Kutlu, Fatih Tornuk

**Affiliations:** ^1^ Department of Food Engineering, Faculty of Chemical and Metallurgical Engineering Yildiz Technical University Istanbul Türkiye; ^2^ Department of Gastronomy and Culinary Arts, Faculty of Fine Arts, Design and Architecture Ankara Medipol University Ankara Türkiye; ^3^ Department of Nutrition and Dietetics, Faculty of Health Sciences Sivas Cumhuriyet University Sivas Türkiye

**Keywords:** biodegradable, cellulose, nanocomposite films, sodium alginate

## Abstract

In the present work, cellulose microfibers (CMFs) isolated from fallen autumn leaves of cherry plum (*Prunus cerasifera pissardii nigra*), white mulberry (*Morus alba)* and plane (*Platanus orientalis*) trees were characterized and used as reinforcement agents in sodium alginate‐based biodegradable films. Fourier transform infrared spectroscopy (FT‐IR) characterization showed that the CMFs were successfully isolated from the leaves with high purity. The extracted CMFs had a particle size ranging from 321.20 nm to 632.26 nm and negative zeta potential values (−27.33 to −21.40). The extraction yield of CMFs ranged from 19.53% to 26.00%. Incorporation of the leaf‐derived CMFs into sodium alginate based films (1%, w:w) increased their tensile strength (from 153.73 to 187.78 MPa) and elongation at break values (from 105.97% to 89.90%) and significantly decreased oxygen (from 121.46 to 75.56 meq kg^‐1^) and water vapor permeabilities (from 2.36 to 1.60 g mm h^−1^ m^−2^ kPa^−1^)(*p* < 0.05). Furthermore, the supplementation of CMFs into the biopolymer matrix had no significant effect on the color (L*: 85.35–85.67; a*: −0.75‐0.71; b*: 4.23–4.94) and moisture content (44.64–48.42%) of the film samples, although the thickness increased (40.33–94.66 μm). Scanning electron microscopy (SEM) images showed that CMFs were homogeneously dispersed in the film matrix. Overall, this study confirms that fallen cherry plum, white mulberry, and plane leaves are valuable sources of CMFs which could be used in the manufacturing of biodegradable nanocomposite films as reinforcement agents.

## Graphical abstract

In this study, cellulose microfibers isolated from fallen leaves were characterized and then used as filler agents in biodegradable sodium alginate based films. Homogeneous film structures were obtained and film properties were improved.

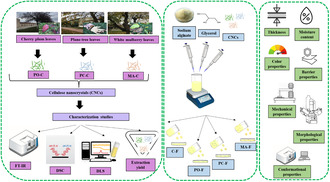

## 1 INTRODUCTION

The rapid growth of the global population has resulted in an increase in waste generation in the world. Especially, as a result of increasing plastic production from the second World War, plastic waste has constituted a significant portion of overall waste, which has brought a significant environmental problem. In order to prevent increasing environmental pollution and to meet the constant need for energy, the trend towards renewable and sustainable energy sources has increased in recent years. All organic matter from both plants and animals is called as biomass. Agricultural crops, algae, animal wastes, aquatic plants, food processing wastes, municipal solid wastes, wood and wood wastes, and their waste by‐products are all examples of biomass resources (Kalak, 2023; Mahongnao et al., 2023; Sadh et al., 2023).

Lignocellulosic plant biomass is a naturally occurring, readily available source of raw materials that are commonly used for energy generation and to produce several useful materials (Awasthi et al., 2023). Lignocellulose is a complex polymer which is composed of three carbohydrates, namely cellulose, hemicellulose and lignin (Dong et al., 2023). Depending on the plant species, the amount of cellulose, hemicellulose, and lignin can vary. Due to some diversities in their chemical structure, they can show distinct chemical reactivities (Perez et al., 2002). Cellulose is a polysaccharide with a crystalline structure that is insoluble in water (Zhang et al., 2023). It is made up of D‐glucose (Dextrose) units connected by β‐(1,4) glycosidic linkages (Kalidas & Sangaranarayanan, 2023). It is a natural, renewable and biodegradable structural biopolymer that has been widely used in many areas, especially in the packaging sector, due to its remarkable attributes such as being cost‐effective and abundant in nature (Kian et al., 2017; Liu et al., 2006). The fact that cellulose is obtained in different forms makes it suitable for use in various fields. Also, cellulose is the most prevalent renewable organic polymer in the biosphere, with an estimated annual output of more than 75 billion tons (Ferrer et al., 2017). Furthermore, nanocellulose has a number of distinguished properties including high stiffness and Young's modulus (up to 134 GPa) and high tensile strength (TS) (up to 7.5 GPa) as well as high aspect ratio, being light weight, low coefficient of thermal expansion, and high surface area/volume ratio (Chen et al., 2011; Tan et al., 2015).

Fallen leaves are classified as forestry wastes/residues which contain high amounts of lignocellulosic material (Cui et al., 2010; Yang et al., 2019). They can offer many advantages such as renewability, high abundance and cheapness (Chanthavong et al., 2024). Although they are widely available, their use is still limited (Tezcan & Atıcı, 2017). A number of utilization routes such as biogas production, manufacture of green flexible graphene–inorganic‐hybrid micro‐supercapacitors, food alternatives, use as adsorbent for removal of toxic agents, and extraction of active compounds have been suggested in the literature (D' Silva et al., 2022; Guo et al., 2020; Kong et al., 2015; Le et al., 2022; Liew et al., 2011; Park et al., 2022; Takahashi & Kaji, 2001; Yakefu et al., 2018; Yang et al., 2019; Yang & Wang, 2021). In addition, several studies have also been performed for isolation of cellulose from leaves of mengkuang, doum (*Chamaerops humilis*), pineapple and ficus trees (Abdul Karim et al., 2022; Fardioui et al., 2016; Reddy et al., 2013; Reddy et al., 2016; Santos et al., 2013; Sheltami et al., 2012). To the best of our knowledge, the isolation and characterization of cellulose microfibers (CMFs) from cherry plum (*Prunus cerasifera pissardii nigra*), white mulberry (*Morus alba*) and plane (*Platanus orientalis*) trees and their application in the biodegradable film production has not been investigated in the literature. Hence, this research aimed to extract and characterize CMFs from fallen leaves of cherry plum, white mulberry, and plane trees. Additionally, it sought to explore their impact as reinforcement agents on the properties of sodium alginate‐based films.

## 2 MATERIALS AND METHODS

In this study, CMFs were first isolated from the selected fallen leaves and characterized using DLS, FTIR and DSC. Then, these fibers were used as reinforcement agents in sodium alginate based biodegradable films. Thickness, moisture content, color, barrier, mechanical, morphological and conformational properties of the resulting film samples were analyzed.

### 2.1 Materials

Glycerol (Merck, Germany), sodium salt of alginic acid (Sigma, Germany), microcrystalline cellulose (MCC, Tito, Türkiye), and cellulose fiber (Katki Dunyasi, Türkiye) were obtained. The reagents used in the current study were in analytical grade and purchased from Merck (Darmstadt, Germany) or Sigma (Germany).

### 2.2 Collection and preparation of fallen leaves

**FIGURE 1:**
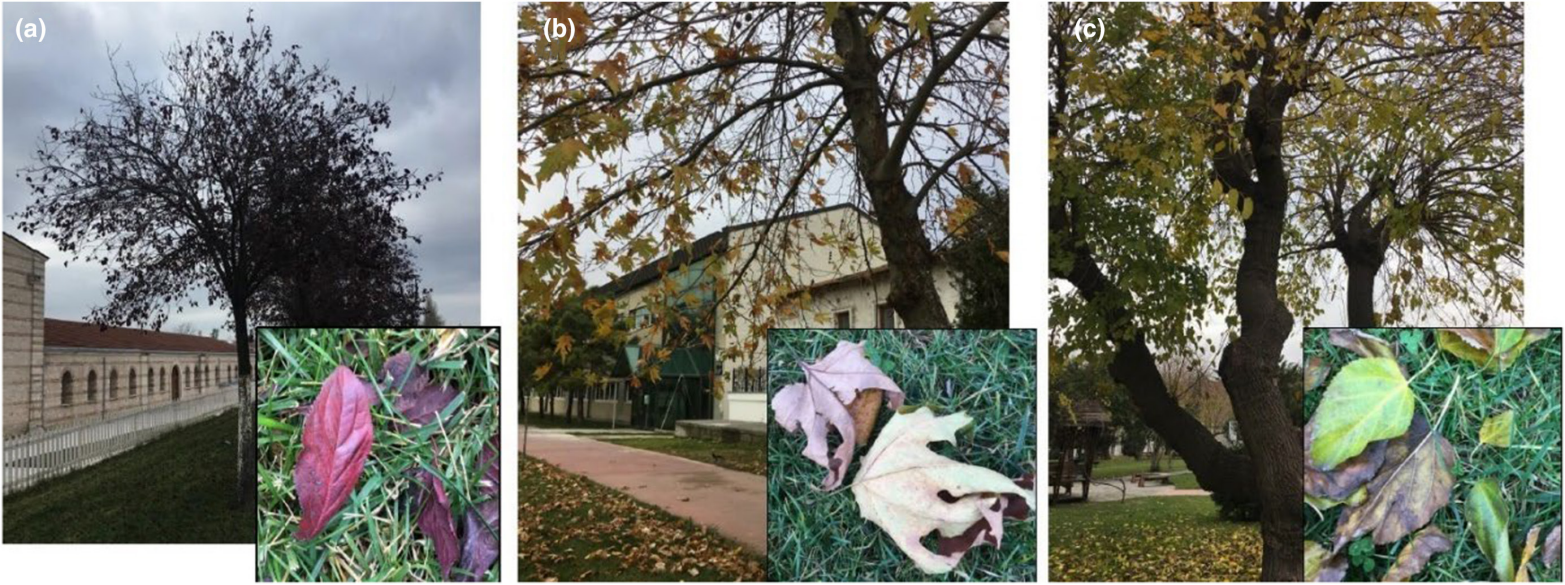
Images of leaves used for CMFs extraction (a) cherry plum (*Prunus cerasifera pissardii nigra*) leaves, (b) plane tree (*Platanus orientalis*) leaves, and (c) white mulberry (*Morus alba*) leaves.

Sufficient amounts of fallen leaves from cherry plum (*Prunus cerasifera pissardii nigra*) (Figure 1a), oriental plane (*Platanus orientalis*) (Figure 1b), and white mulberry (*Morus alba*) (Figure 1c) trees found in Yildiz Technical University Davutpaşa Campus (Istanbul, Türkiye) were collected during the autumn period (September–October) of 2021 and transferred to the laboratory in plastic bags. To eliminate dirts and impurities, the leaves were washed with distilled water for 2 min and then dried at room temperature overnight. Then, the dried leaves were ground with a grinding machine (Tefal 8100.31 coffee grinder, France) to obtain leaf powders. The ground leaves underwent Soxhlet extraction with toluene: ethanol (2:1, w:w) for 6 h for dewaxing. The dewaxed samples were then dried in an oven (Memmert UF‐110, Germany) at 40°C (Morán et al., 2008).

### 2.3 Extraction of CMFs

**FIGURE 2:**
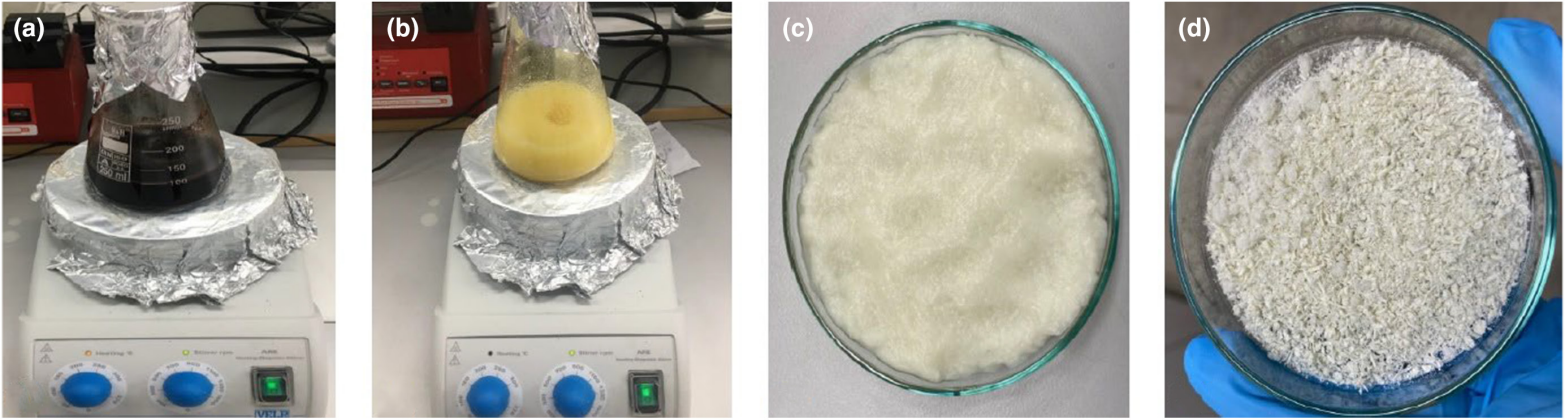
Experimental procedure photos for CMFs extraction (a) alkali‐treated samples, (b) bleached samples, (c) bleached samples washed with pure water, and (d) lyophilized CNCs samples.

CMF extraction was performed according to the protocol described by Sheltami et al. (2012). The images illustrating each extraction step are given in Figure 2. For this purpose, approximately 5 g of dewaxed leaf sample was treated with 4% NaOH for hemicellulose removal at 125°C and 1100 rpm for 2 h using a magnetic stirrer (Heidolph MR 3001, Germany), with two replications . The sample was then separated from the solvent by filtration using coarse filter paper. Afterwards, bleaching was performed using 1.7% sodium chlorite (NaClO_2_) and 0.5 mL of acetic acid (CH_3_COOH) mixture at 125°C for 3 h to purify the CMF. The CMFs obtained from the three different tree leaves were then washed with distilled water and lyophilized (Martin Christ, Beta 1–8 LSC plus, Osterode am Harz, Germany). The samples were coded as PO‐C, MA‐C, and PC‐C based on their origin of oriental plane, white mulberry, and cherry plum leaves, respectively.

### 2.4 Characterization of CMFs

The resulting CMFs were characterized for their molecular, morphological and thermal properties and their particle size distribution and zeta potential properties were analyzed. Commercial microcrystalline cellulose (MCC) and cellulose fibers were also tested for comparison.

#### 2.4.1 Molecular characterization by FTIR

The samples were pelleted using a manual hydraulic press device and FTIR analysis was performed with a wavelength range of 400–4000 cm^−1^ with a resolution of 4 cm^−1^ using the FTIR instrument (Bruker Tensor 27 + HTSXT, Bremen, Germany) (Kutlu et al., 2020).

#### 2.4.2 Differential scanning calorimetry (DSC)

Thermal characterization of the CMFs was performed using a DSC device (DSC, Q100, TA Instruments Inc., New Castle, DE, USA) according to the method described by Almasi et al. (2010). For this aim, ~4 mg of CMF was weighed, placed in an aluminum hermetic pan and, heated in a nitrogen atmosphere starting from 40°C to 400°C at a heating rate of 10°C min^‐1^ (Akman et al., 2023).

#### 2.4.3 Particle size and surface charge (ζ‐potential) measurements by dynamic light scattering (DLS)

Both the average particle size and stability measurements were performed with DLS measurements employing a zetasizer (Nano ZSP, Malvern Instruments Corp., Worcestershire, U.K.) at 25°C, following the method described by Naduparambath and Balan (2018). For this aim, dilute aqueous CMF suspensions were prepared and analyzed after ultrasonication (Daihan, WUC‐D10H, North Korea) for 5 min.

#### 2.4.4 Calculation of CMF yield

The percent CMF yields were calculated on a dry weight basis by dividing the weight of the initial leaf material by the weight of the final CMF powder (Liu et al., 2006).

### 2.5 Production of CMF loaded sodium alginate films

The production of sodium alginate based films was performed based on the former protocol described by Mahcene et al. (2021). Firstly, CMF (1% of the sodium alginate, w:w) was suspended in 100 mL of distilled water and stirred at 1500 rpm in a magnetic stirrer (Heidolph MR 3001, Germany) at room temperature for 24 h, followed by sonication for 3 min. Subsequently, sodium alginate (2.5%, w/v) and glycerol (3%, w/v) were added to the CMF suspension and the mixture was kept in a shaking water bath (Memmert Model SV 1422, Schwabach, Germany) at 80°C for 2 h to ensure complete dissolution of the biopolymer. Then, the resulting film solution was poured into plastic Petri dishes and dried in a fan oven at 40°C for 24 h. The biodegradable films obtained were labeled as PO‐F, MA‐F, and PC‐F, corresponding to the CMF origin of oriental plane, white mulberry, and cherry plum, respectively. A control film sample without CMF filler (C–F) was also fabricated.

### 2.6 Characterization of CMF loaded sodium alginate films

#### 2.6.1 Film thickness

Film thickness measurements were performed using a digital micrometer (Mitutoyo, IP65 Coolant Proof, Japan) with an accuracy of 1 μm according to the method described by Memiş et al. (2017). For this aim, at least five random measurements were made at various locations on the films and the average film thickness values were calculated.

#### 2.6.2 Moisture content

The gravimetric method described by Dick et al. (2015) was used for the determination of the moisture content of the films. For this purpose, the percent moisture value of each CMF loaded sodium alginate films was calculated after drying approximately 1–2 g of film samples in an oven (Memmert UF‐110, Germany) at 105°C for 24 h.

#### 2.6.3 Color and film transparency

The methodology proposed by Jafarzadeh et al. (2016) was used for measurement of color properties (L* (white‐black), a* (red‐green) and b* (blue‐yellow)) of the films using a portable colorimeter (CR‐400, Minolta Camera Co., Osaka, Japan). The film samples were placed on the standard white plate and the measurement was done at five different points of each film sample. Mean ΔE* (total color difference), Δa*, Δb*, and ΔL* values for film samples were also calculated using the following formula:

(1) $$∆E^{*}=\sqrt{{\left(∆a^{*}\right)}^{2}+{\left(∆b^{*}\right)}^{2}+{\left(∆L^{*}\right)}^{2}}$$

(2) $$∆a^{*}=a_{1}^{*}-a_{0}^{*}$$

(3) $$∆b^{*}=b_{1}^{*}-b_{0}^{*}$$

(4) $$∆L^{*}=L_{1}^{*}-L_{0}^{*}$$

Film transparency (*T*) was measured using a UV–vis spectrophotometer (Shimadzu, UV‐1800, Japan) at 600 nm based on the method described previously by (Park et al., 2004). For this purpose, the film samples were cut into rectangular pieces and placed in the spectrophotometer cuvette before measurement of their absorbance. *T* was calculated using the following equation:

(5) T=Abs600x

where *T, Abs*_*600*_ and *x* are transparency, absorbance value at 600 nm and film thickness, respectively.

#### 2.6.4 Barrier properties

Water vapor permeability (WVP) of the CMF loaded sodium alginate‐based films was gravimetrically analyzed previously noted by ASTM (2007). Firstly, test tubes containing 2 g of silica gel were put in an oven 105°C for 24 h and then covered with the film samples. The sealed tubes were then kept in a water‐filled desiccator at 25°C for 24 h. The weight of the tubes was measured at 0^th^, 1^st^, 2^nd^, 3^rd^, 4^th^, 23^rd^ and 24^th^ h of the storage. The WVP of the CMF loaded sodium alginate based films was calculated using the following equation:

(5) $$\mathrm{WVP}=\frac{w}{t}x\frac{x}{∆\mathrm{PxA}}$$

where *x*, ∆ *P*, *A* were the mean film thickness (μm), the relative pressure difference (kPa) at 25°C and the film surface area, respectively. The *w*/*t* was determined by the measurement of the absorbed water by the system at the steady state.

In order to determine the oxygen transmission rate (OTR) of CMF loaded sodium alginate films, the peroxide values of the samples were measured using the methodology proposed by Memiş et al. (2017). Briefly, the tubes (25 mL in volume) were filled with 15 mL of antioxidant‐free sunflower oil and then covered with the film samples. Then, they were left in an oven at 60°C for 10 days. Finally, sodium thiosulfate (Na_2_S_2_O_3_) titration was used to determine the peroxide value of the oil samples.

#### 2.6.5 Mechanical properties

Employing a texture analyzer (TA HD Plus, Stable Micro Systems, UK) equipped with a 5 kg of load cell, the mechanical characteristics of the CMF loaded sodium alginate films were measured (Memiş et al., 2017). The films were cut into rectangles (2 cm × 7 cm) and fastened to the tensile grips. The initial distance between the grips of the probe was set to 50 mm, and the test was performed with a strain rate of 10 mm s^‐1^. The TS and elongation at break (EB) values were recorded.

#### 2.6.6 SEM

A field emission SEM (FE‐SEM, Quanta FEG 250, FEI, USA) tool was used to observe the surface morphologies of the sodium alginate based films. For this purpose, the film samples were cut into small pieces and placed directly on a sample holder after being coated with gold. The acceleration voltage was set to 5.0 kV and, the SEM images were magnified 5, 10, and 20 times (Subaşı‐Zarbaliyev et al., 2023).

#### 2.6.7 FTIR spectroscopy

The molecular characterization of the film samples was performed using an FTIR instrument (Bruker Tensor 27 + HTSXT, Germany) within the wavelength range of 400–4000 cm^−1^. The film samples were cut into squares (2 cm × 2 cm) and placed directly into the beam zone (Memiş et al., 2017).

### 2.7 Statistical analysis

Statistical analysis was performed using MSO for Microsoft® Excel® Microsoft 365 (Version 2205 Build 16.0.15225.20172). Mean values from at least three parallels of triplicate experiments were calculated. Moreover, significant differences between the means were analyzed using one‐way analysis of variance (ANOVA) followed by Duncan's multiple comparison test, with a significance level of 95%.

## 3 RESULTS AND DISCUSSION

### 3.1 Characterization of CMFs

#### 3.1.1 FTIR spectroscopy

**FIGURE 3:**
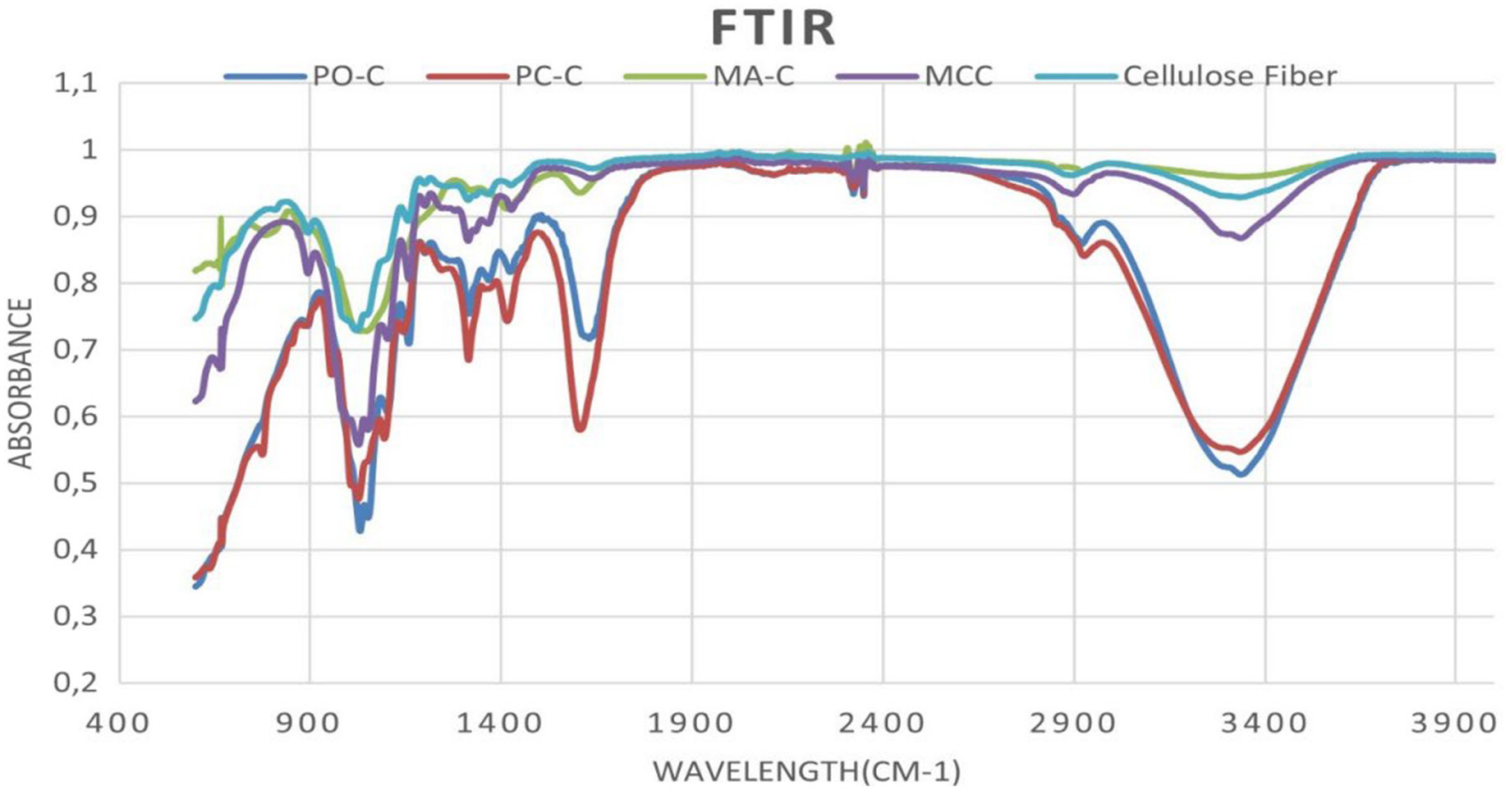
FTIR spectra of the CMFs. Cellulose fiber, Commercial cellulose fiber; MCC, Commercial microcrystalline cellulose; MA‐C, CMF extracted from white mulberry tree (*Morus alba*) leaves; PC‐C, CMF extracted from plane (*Platanus orientalis*) tree leaves, PO‐C, CMF extracted from cherry plum (*Prunus cerasifera pissardii nigra*) tree leaves.

Given the unique wavelengths at which molecular functional groups vibrate, FTIR spectroscopy is a powerful analytical method for identifying and elucidating the structures of chemical components in samples. In the present work, FTIR spectroscopy was used to identify the functional groups and bonding types present in PO‐C, MA‐C and PC‐C, as well as commercial cellulose (MCC and cellulose fiber) samples, as shown in the spectra given in Figure 3. As shown in the figure, although peak intensities varied depending on the CMF type, PO‐C, MA‐C, and PC‐C generally exhibited vibrational frequencies similar to those of the commercially available MCC and cellulose fiber. The FTIR spectra revealed that peak intensities of PO‐C, PC‐C were lower compared to the MA‐C, MCC and cellulose fiber. The spectra of all samples showed two distinct absorbance zones in the wavelength ranges from 2800 cm^−1^ to 3500 cm^−1^ and from 500 cm^−1^ to 1700 cm^−1^. Peaks around 3440–3300 cm^−1^ were attributed to the stretching of‐OH groups (Amara et al., 2023; Kian et al., 2017). The absence of absorbance around 1726 cm^−1^ and 1533 cm^−1^ indicates the lack of acetyl and uronic ester groups of hemicelluloses, ester bonds of lignin, and C=C stretching of the aromatic ring of lignin (Trilokesh & Uppuluri, 2019), demonstrates the effective removal of lignin and hemicellulose from the samples. The vibrations around 1642 cm^−1^ reflected to the adsorbed water (Alemdar & Sain, 2008). Moreover, the observed peaks at 1430 cm^−1^ could be corresponding to CH_2_ bending vibration, which represented the crystallinity of the sample (Kian et al., 2017). Furthermore, the weak peak of the C–H asymmetric deformations were around 1385 cm^−1^ (Alemdar & Sain, 2008). Additionally, the peaks located around 1060–1050 cm^−1^ and 895 cm^−1^ (β‐glycosidic linkage vibration) in the spectra of samples were representative of C‐H stretching vibration of C‐O and the typical characteristic structure of the cellulose component (Johar et al., 2012). Abdul Rahman, Chieng, Ibrahim, and Abdul Rahman et al. (2017) reported that the overall peak intensities increased with the increase in the percentage of cellulosic components. Based on this information, it could be interpreted that among the extracted CMF samples, purity of MA‐C was higher than those of PO‐C and PC‐C. In addition, the peaks near 750 and 710 cm^−1^ were attributed to the monoclinic (I_β_) and triclinic (I_α_) cellulose (Jahan et al., 2011) while the highest proportion of I_β_ was determined in MA‐C, followed by PO‐C and PC‐C.

#### 3.1.2 DSC

**FIGURE 4:**
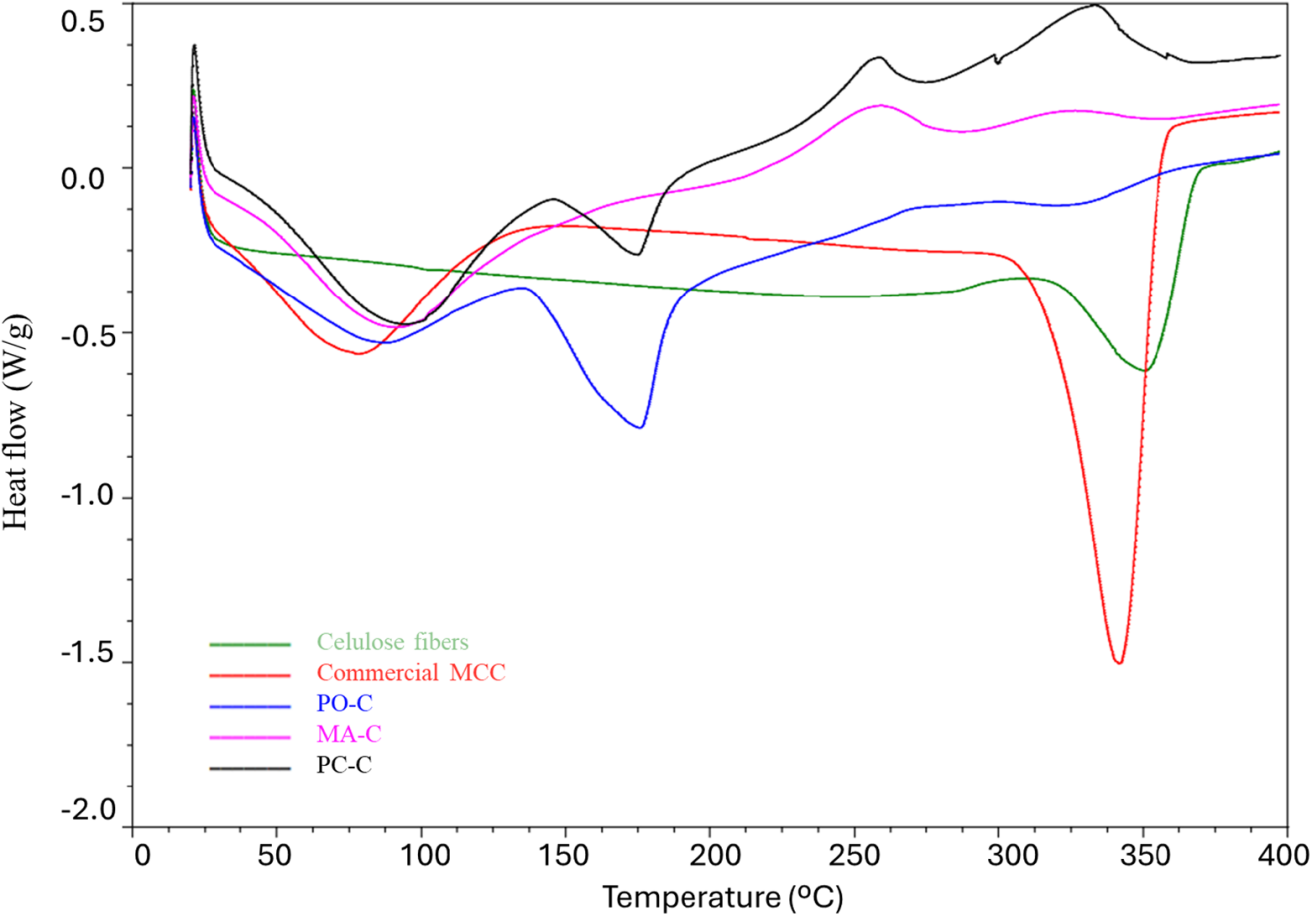
DSC thermograms of CMFs as well as commercial MCC and cellulose fibers. Cellulose fiber, Commercial cellulose fiber; MCC, Commercial microcrystalline cellulose; MA‐C, CMF extracted from white mulberry tree (*Morus alba*) leaves; PC‐C, CMF extracted from plane (*Platanus orientalis*) tree leaves, PO‐C, CMF extracted from cherry plum (*Prunus cerasifera pissardii nigra*) tree leaves.

DSC analysis was performed to compare the thermal behavior and thermal stability of PO‐C, MA‐C, PC‐C. DSC thermograms of CMFs as well as commercial MCC and cellulose fibers are presented in Figure 4. According to the DSC measurements, all CMF samples exhibited both endothermic and exothermic peaks. The first peak (endothermic) was related to the evaporation of the free water while the second one (exothermic) represented the thermal decomposition. Among the tested samples, MA‐C showed the highest endothermic temperature (103.74°C), followed by PO‐C (102.81°C) while PC‐C exhibited the lowest (69.42°C) as presented in Table 1. From the findings, it was clear that PC‐C required the lowest energy for the evaporation of the bound water content than the other CMF samples. These findings were consistent with those reported in other studies (Bosenbecker et al., 2022) and supported the idea that the initial thermal transition maintained around 100°C possibly due to the absorption of thermal energy. Additionally, thermal degradation temperatures (T_d_) of the CMFs were between 333.33 and 350.38°C (Table 1) while the highest and lowest T_d_ values were measured at PO‐C and MA‐C, respectively. The result of this study was in accordance with the findings of Matebie et al. (2021) who studied CNCs extracted from brewer's spent grain and reported that extracted raw cellulose and CNCs had high thermal stability with T_d_ values of 310 and 335°C, respectively. Similarly, Morán et al. (2008) reported that the peak values for cellulose from sisal fiber were in the range of 76.70–84.91°C and 318–346.6°C. Overall, the results showed that among the tested samples, PO‐C had the highest thermal stability due to the requirement of higher energy absorption for degradation.

**TABLE 1:** Endothermic and exothermic temperatures of the CMFs obtained by DSC.

Sample name	Endothermic temperature (°C)	Exothermic temperature (°C)
PC‐C	69.42	340.79
PO‐C	102.81	350.38
MA‐C	103.74	333.33

Abbreviations: MA‐C, CMF extracted from white mulberry tree (*Morus alba*) leaves; PC‐C, CMF extracted from plane (*Platanus orientalis*) tree leaves, PO‐C, CMF extracted from cherry plum (*Prunus cerasifera pissardii nigra*) tree leaves.

#### 3.1.3 Particle size, PDI, and ζ‐potential

Average particle size, polydispersity index (PDI) and ζ‐potential values of PC‐C, PO‐C and MA‐C are summarized in Table 2. Average particle size is an important index since it directly affects the stability. Larger particles tend to agglomerate more readily than smaller ones, which causes to quick sedimentation. In decreasing order, the average particle size values of the PC‐C, PO‐C and MA‐C were 632.26 nm, 398.90 nm and 321.20 nm, respectively, with no significant difference between the size values of PO‐C and MA‐C (*p* > 0.05). Similar average particle size values were reported for CNCs from brewery's spent grain by‐product (309.4 nm) (Matebie et al., 2021), spherical CNCs from jackfruit peel (346 nm) (Trilokesh & Uppuluri, 2019) and CNCs from rice husk (700 nm) (Lu & Hsieh, 2012). Differences in particle size values could be related to the variations in the raw material and CMF hydrolysis conditions (e.g. acid type, acid concentration, hydrolysis duration, hydrolysis temperature, liquid/solid ratio) (Matebie et al., 2021).

**TABLE 2:** Average particle size, polydispersity index, stability, and extraction yield of the CMFs.

Sample name	Particle size (nm)	PDI	ζ‐Potential (mV)	Extraction yield (%)
PC‐C	632.26 ± 72.70^a^	0.48 ± 0.10^ab^	−21.40 ± 1.81^a^	19.53 ± 2.20^b^
PO‐C	398.90 ± 79.80^b^	0.41 ± 0.10^*c*^	−27.33 ± 1.20^b^	26.00 ± 2.80^a^
MA‐C	321.20 ± 73.70^b^	0.44 ± 0.10^*bc*^	−24.26 ± 0.90^ab^	24.16 ± 2.30^ab^

*Note*: ^a‐c^Different letters in the same column indicated statistically significant differences (p < 0.05).

Abbreviations: MA‐C, CMF extracted from white mulberry tree (*Morus alba*) leaves; PDI, Polydispersity index, PC‐C, CMF extracted from plane (*Platanus orientalis*) tree leaves, PO‐C, CMF extracted from cherry plum (*Prunus cerasifera pissardii nigra*) tree leaves.

The ratio of the size deviation (or width of the distribution) to the mean particle diameter is the standard definition of the PDI. If the PDI is zero, there is no size fluctuation, however, a PDI close to 1 implies a significant particle size variation. Also, PDI values lower than 0.3 mean that the particles are largely monodisperse. The PDI values (Table 2) of PC‐C, MA‐C and PO‐C were 0.48, 0.44, and 0.41, respectively, indicating moderate polydispersity. Although there were significant (*p* < 0.05) differences in the PDI levels of the samples, the the samples had similar polidispersity, suggesting that the effect of CMF source on PDI was limited.

Analysis of the CMFs in aqueous suspensions is highly essential in order to determine their colloidal stability. Also, the stability evaluation of CMF dispersion in water can be performed by interpreting their ζ‐potential values (Bahloul et al., 2021). As shown in Table 2, ζ‐potential of CMF samples had negative values. PO‐C had the highest negative ζ‐potential (−27.33 mV), followed by MA‐C (−24.26 mV) and PC‐C (−21.40 mV), respectively. Values of ζ‐potential lower than −25 mV or more than 25 mV means better stability due to electro‐static/charge repulsion or attraction between particles, indicating that PO‐C generated more stable colloidal dispersions and had better electrostatic stabilization property than MA‐C and PC‐C. Similarly, Elanthikkal et al. (2010) extracted CMFs from banana plant wastes and reported ζ‐potential values ranging from −41.32 to −20.72 mV depending reaction conditions of the acid hydrolysis. Similar ζ‐potential values (−25.9‐(−20.6) mV) were also reported for CNCs obtained from roselle‐derived microcrystalline cellulose (Kian et al., 2018). In previous research, ζ‐potential values ranging from −13.8 to −32.7 mV were observed for the CNCs obtained from pineapple peel at different extraction periods (Madureira et al., 2018). In another study, Bahloul et al. (2021) extracted micro and nano‐sized cellulose from the eggplant plant residue and found their ζ‐potential values between –39.4 and –28.7 mV. Reversely, higher negative ζ‐potential values were reported for CNCs from bamboo fibers (−60‐(−37.7) mV) (Brito et al., 2012), doum leaves (−31 mV) (Fardioui et al., 2016) and sugar palm fibers (−61.50 mV) (Ilyas et al., 2018).

#### 3.1.4 CMF yield

The main factors affecting CMF yield include the type of raw material, the acid‐to‐raw material ratio, the extraction temperature, and the extraction duration (Bondeson et al., 2006). In this study, extraction yields of PC‐C, MA‐C and PO‐C were 19.53%, 24.16%, and 26.00%, respectively (Table 2) with the raw material having a significant effect on CMF extraction yield (*p* < 0.05). These findings were in line with the studies on CNCs from bamboo fibers (9%–30%) (Brito et al., 2012), CNCs from red algae waste (21.5%) (El Achaby et al., 2018), cellulose from perennial ryegrass leaves (22.7%–36.8%) (Liu et al., 2006), CNCs from jackfruit peel (20.08%) (Trilokesh & Uppuluri, 2019), and CNCs from roselle‐derived microcrystalline cellulose (12.51%–21.92%) (Kian et al., 2018). Higher extraction yields were also noted for cellulose from Josapine pineapple leaf fiber (70.9%–73.5%) (Fareez et al., 2018), CNCs from mengkuang leaves (28%) (Sheltami et al., 2012), CNCs from sugar palm fibers (29%) (Ilyas et al., 2018) and CMF from olive pomace (57.63%) (Amara et al., 2023).

### 3.2 Characterization of CMF loaded sodium alginate based films

#### 3.2.1 Film thickness

The thickness values of C‐F, PC‐F, PO‐F and MA‐F film samples are given in Table 3. As seen, MA‐F had the highest thickness value (94.66 μm), followed by PC‐F (90.33 μm), PO‐F (84.33 μm), and C‐F (40.33 μm), respectively. The incorporation of CMF into the sodium alginate films significantly increased the thickness values (*p* < 0.05), while the origin of CMF was insignificant (*p* > 0.05). Similar observations were also reported by Shankar and Rhim (2016) and Doh et al. (2020).

**TABLE 3:** Physical (thickness and moisture content), barrier (PV and WVP) and mechanical properties (TS and EB) of CMF loaded biodegradable film samples.

Film samples	Thickness (μm)	Moisture content (%)	PV (meq/kg)	WVP (g mm h^−1^ m^−2^ kPa^−1^)	TS (MPa)	EB (%)
C‐F	40.33 ± 0.04^a^	48.42 ± 1.71^a^	121.46 ± 9.81^*a*^ ^a^	2.36 ± 0.23^a^	153.73 ± 14.82^a^	105.97 ± 2.22^a^
PC‐F	90.33 ± 0.09^b^	44.64 ± 3.12^a^	91.26 ± 11.89^bd^	2.09 ± 0.16^a^	187.78 ± 11.25^a^	105.75 ± 4.86^a^
PO‐F	84.33 ± 0.08^a^	48.13 ± 1.73^a^	74.72 ± 3.18^cd^	1.60 ± 0.03^b^	157.10 ± 13.17^bc^	96.52 ± 2.63^b^
MA‐F	94.66 ± 0.09^b^	47.39 ± 2.07^a^	75.55 ± 3.93^a^	2.33 ± 0.20^a^	180.49 ± 15.64^a^	89.90 ± 0.66^a^

Abbreviations: C‐F, Neat sodium‐alginate film (control), EB, Elongation at break, MA‐F, MA‐C incorporated sodium alginate film, PC‐F, PO‐C incorporated sodium alginate film; PO‐F, PC‐C incorporated sodium alginate film; PV, Peroxide value; TS, Tensile strength; WVP, Water vapor permeability.

^a‐d^ Different letters in the same column indicated statistically significant differences (p < 0.05).

#### 3.2.2 Moisture content

The moisture contents of the sodium alginate based films ranged from 44.64% to 48.42%i with  no significant difference (*p* > 0.05) between the samples (Table 3). In a related work conducted by Doh et al. (2020), addition of CNCs derived from kombu and sargassum into the sodium alginate films resulted in a significant decrease in the moisture content. Abdollahi et al. (2013) also reported a decrease in the moisture content of alginate films with the supplementation of CNCs into the polymer matrix.

#### 3.2.3 Barrier properties

PVs of the CMF reinforced sodium alginate based films, showing their oxygen barrier properties, are given in Table 3. The decrease in PV values indicates that the incorporation of CMF significantly (*p* < 0.05) decreased the oxygen permeability of the biopolymer films. This means that the incorporation of PO‐C, PO‐C and MA‐C into the polymer matrix resulted in decrease in the oxygen permeability of 24.86, 38.48, and 37.67% , respectively, as compared to the control film. Similar trends were also reported by Luzi et al. (2016) and Doh et al. (2020).

The WVP values of the biodegradable films are presented in Table 3. As expected, the control film exhibited the lowest water vapor barrier ability. The reduction in WVP values with CNF incorporation was likely due to the increased tortuosity of the pathways within the polymer matrix (Azeredo et al., 2010). This was also evidence of well‐dispersion of CMF in the polymer matrix (Abdollahi et al., 2013). Similar results were reported by Azeredo et al. (2009) and Khan et al. (2012) with the incorporation of cellulose into film materials.

#### 3.2.4 Mechanical properties

The mechanical properties (EB and TS) of C‐F, PC‐F, PO‐F and MA‐F films are given in Table 3. As expected, TS values of biopolymer films were significantly improved by the addition of fallen leaf‐derived CMFs. The percent increasesn i TS values were 22.14%, 2.19%, and 17.41% as affected by the reinforcement of the sodium alginate films with PC‐F, PO‐F and MA‐F, respectively, as compared to the neat film sample. The increase in TS with CMF addition could be attributed to the formation of new chemical interactions between polymer chains in the film matrix (Wang et al., 2017). These findings were in accordance with the reports of Abdollahi et al. (2013), Shankar and Rhim (2016) and Doh et al. (2020).

As shown in Table 3, the EB values of the sodium alginate‐based films were negatively influenced by the addition of the CMFs. The EB levels ranged from 89.90% to 105.97%, with the control film exhibiting the highest elongation rate. No significant differences were found between C‐F and PC‐F (*p* > 0.05). Also, there was no significant difference between EBs of PO‐F and MA‐F (*p* > 0.05). These results revealed that the addition of the CMFs as fillers decreased the flexibility of the film, likely due to the formation of strong interactions between the film components, causing the restriction of polymer chain mobility, thereby lowered the EB values (Khan et al., 2012). An other possible explanation could be the rigid nature of the filler (Abdollahi et al., 2013). Overall, the results were in accordance with existing literature (Abdollahi et al., 2013; Azeredo et al., 2010; Doh et al., 2020; Khan et al., 2012; Wang et al., 2017).

#### 3.2.5 Color and transparency

Color is an important parameter in food packaging because it affects how films look, which can have a direct impact on consumer acceptability during marketing (Li et al., 2020). The color parameters, namely L*, a*, b*, and *ΔE* (total color difference), are presented in Table 4. The L* and a* values of the biopolymer films ranged from 85.26 to 85.67 and from −0.75 to 0.72, respectively. As seen, the addition of fallen leaf derived CMFs did not make any significant influence (*p* < 0.05) on L*, a* and b* values of the films. These findings were in line with the literature (Shankar & Rhim, 2016; Wang et al., 2017). Furthermore, ΔE* values of film samples were between 0.00 and 1.53, indicating that the color difference between the control and CMF‐loaded film samples were negligible since the ΔE* values lower than 3 are generally not detectable by the human eye (Yavuz et al., 2022). Similarly, Wang et al. (2017) reported that the color of the CNC incorporated films was not significantly influenced by the filler type and concentration.

**TABLE 4:** Color properties (L*, a*, b*, ΔE*) and *T* values of CMF loaded biodegradable film samples.

Film samples	L*	a*	b*	ΔE*	*T*
C‐F	85.35 ± 0.33^a^	0.71 ± 0.18^a^	4.94 ± 0.76^a^	0.00	1.24 ± 0.06^a^
PC‐F	85.51 ± 0.27^a^	−0.75 ± 0.16^a^	4.89 ± 0.71^a^	1.47	2.16 ± 0.14^b^
PO‐F	85.26 ± 0.51^a^	−0.71 ± 0.03^a^	4.79 ± 0.22^a^	1.43	5.25 ± 0.35^a^
MA‐F	85.67 ± 0.15^a^	−0.61 ± 0.04^a^	4.23 ± 0.29^a^	1.53	1.23 ± 0.06^c^

Abbreviations: C‐F, Neat sodium‐alginate film (control); MA‐F, MA‐C incorporated sodium alginate film; PC‐F, PO‐C incorporated sodium alginate films; PO‐F, PC‐C incorporated sodium alginate film; *T*, Transparency; ΔE*, total color difference.

^a‐c^ Different letters in the same column indicated statistically significant differences (*p* < 0.05).

The *T* values, as presented in Table 4, ranged from 1.23 to 5.25. The C‐F and MA‐F film samples had the lowest *T* values (*p* < 0.05), indicating higher transparency. In contrast, the *T* values of the PC‐F and PO‐F reinforced film samples were higher than those of the control film, suggesting that the incorporation of cherry plum and oriental plane derived CMFs affected the transparency. A related study found that *T* values of the film samples were reduced by the addition of cellulose fibers or cellulose nano‐whiskers (Wang et al., 2017). These findings also indicated a homogeneous film structure since *T* values lower than 5 generally suggest good and homogeneous dispersion of nano‐reinforcement agents in the polymer matrix (Abdollahi et al., 2013).

#### 3.2.6 Morphological properties

**FIGURE 5:**
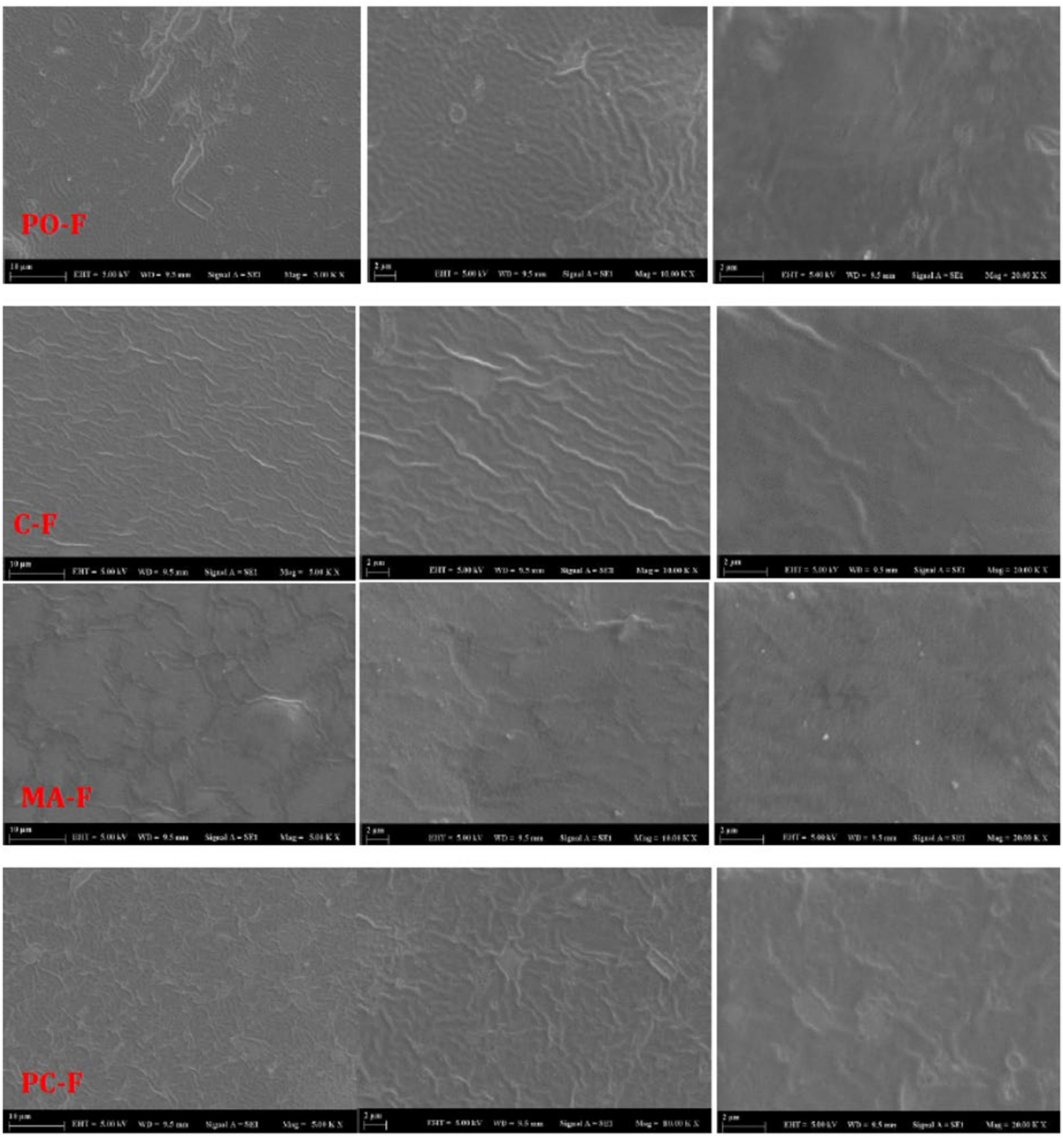
SEM images of CMF incorporated biodegradable film samples. C‐F, Neat sodium‐alginate film (control); PO‐F, PC‐C incorporated sodium alginate film; MA‐F, MA‐C incorporated sodium alginate film; PC‐F, PO‐C incorporated sodium alginate film.

SEM images of film samples are presented in Figure 5. The figures revealed that all film samples had a uniform morphology, with CMFs well and homogeneously dispersed in the biopolymer matrix. Notably, MA‐F had shiny white dots, possibly due to the transversal sections of CMF (Azizi Samir et al., 2005; Khan et al., 2012). The SEM images of the film samples were similar to those reported by other authors (Abdollahi et al., 2013; Chang et al., 2010; Reddy & Rhim, 2014; Wang et al., 2017).

#### 3.2.7 FTIR spectroscopy

**FIGURE 6:**
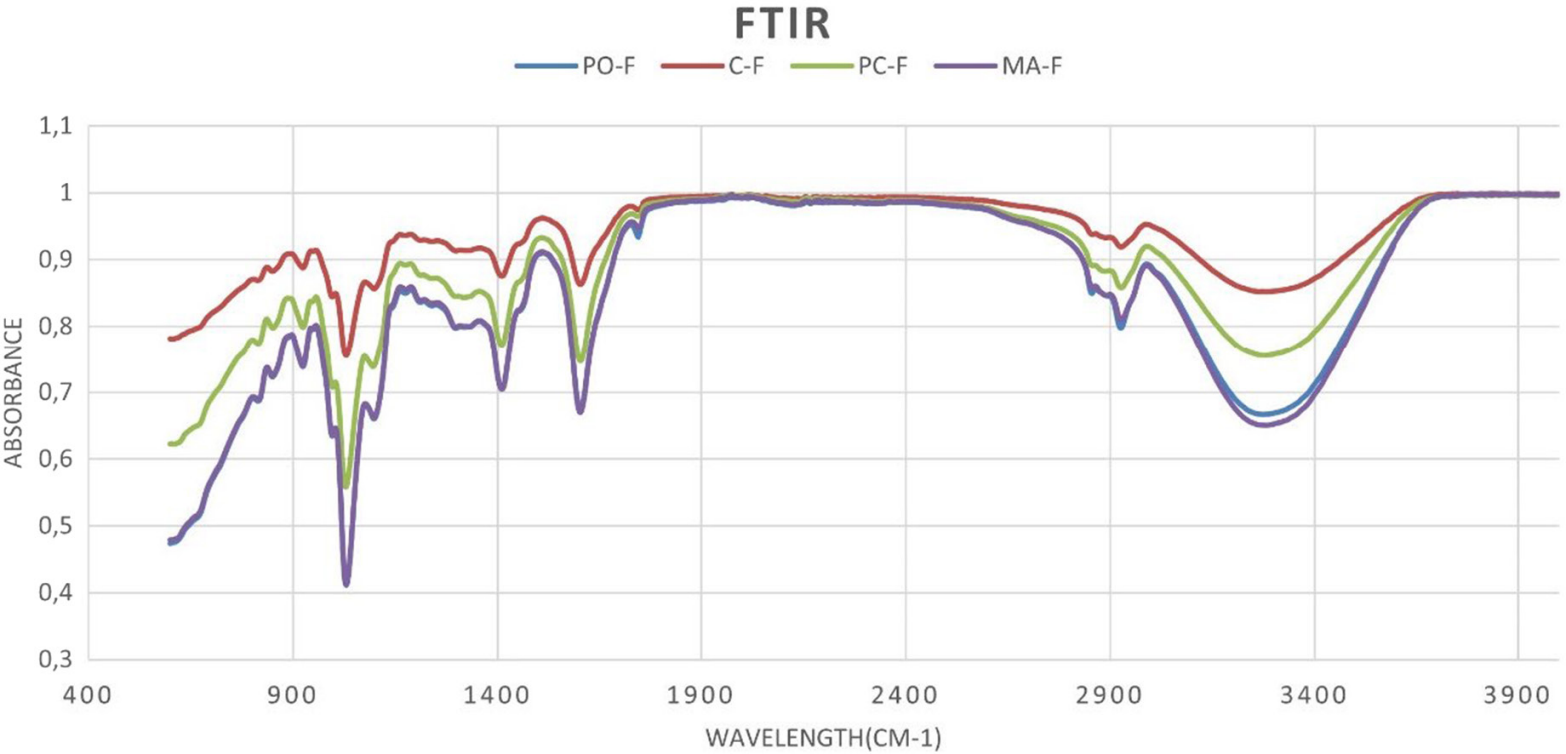
FT‐IR spectra of CMF incorporated biodegradable film samples. C‐F, Neat sodium‐alginate film (control), PO‐F, PC‐C incorporated sodium alginate film, MA‐F, MA‐C incorporated sodium alginate film, PC‐F, PO‐C incorporated sodium alginate films.

FTIR analysis was performed in order to determine the influences of CMF incorporation on the molecular properties of the polymer, with the spectra of the films presented in Figure 6. The sodium‐alginate based film exhibited a spectrum similar to those reported by various researchers (Huq et al., 2012; Wang et al., 2017). The absorption peaks of the sodium alginate film samples were mainly assigned to the O‐H stretching vibrations (3500–3000 cm^−1^) (Doh et al., 2020; Khan et al., 2012), overlapping symmetric and asymmetric C‐H stretching vibrations of aliphatic chains (2930 cm^−1^) (Celebi & Kurt, 2015; Doh et al., 2020), OH stretching vibrations and absorption bands (1595 cm^−1^ and 1412 cm^−1^) (Wang et al., 2017), C‐O, C‐C and ring structures (1160 cm^−1^ and 1055 cm^−1^) (Huq et al., 2012; Wang et al., 2017). Although reinforcement of the films with leaf derived CMFs resulted in some minor differences in the intensities and widths of the peaks, no new peak formation and shifts were observed. Similar observations were also reported by Celebi and Kurt (2015), who incorporated CNCs into chitosan film matrix.

## 4 CONCLUSION

In the present study, CMFs were successfully isolated from three different types of fallen autumn leaves (cherry plum, white mulberry and plane trees) with high yield and purity. The results demonstrated that the CMFs extraced from plane leaves exhibited superior thermal stability, lower PDI and average particle size, and higher colloidal stability as compared to the CMFs from cherry plum and white mulberry. Additionally, these leaf derived CMFs were used as reinforcement agents in sodium alginate based edible film matrix. Incorporation of CMFs into sodium alginate based films increased film thickness while moisture content and color values remained relatively unchanged. Mechanical and barrier properties of the film samples were improved by CMF incorporation. SEM analysis revealed a uniform morphology across all film samples. Similar FTIR spectra of the sodium alginate based films were observed with the incorporation of CMFs. In conclusion, this study showed that fallen leaves could be effectively utilized for CMF recovery to be used as filler agents in sodium alginate based biodegradable films, resulting in improved properties.

## AUTHOR CONTRIBUTIONS

**Sudenur Celik:** Data curation (equal); investigation (lead); methodology (equal); writing ‐ original draft; visualisation. **Gozde Kutlu:** Investigation (equal); writing – original draft (lead); review & editing; visualisation. **Fatih Tornuk:** Conceptualization (lead); project administration (lead); supervision (lead); validation (lead).

## FUNDING INFORMATION

The authors extend their gratitude to Türkiye Bilimsel ve Teknolojik Araştırma Kurumu (TUBITAK) for covering the open‐access publication charges.

## CONFLICT OF INTEREST STATEMENT

All authors declare that there is no conflict of interest in this manuscript.

## ETHICS STATEMENT

There is no ethical declaration.

## Data Availability

Data supporting the findings of this study are available from the corresponding author upon request.
